# Partial cancerous changes eventually developing into superficial spreading cancer over 18 months

**DOI:** 10.3747/co.v16i6.424

**Published:** 2009-12

**Authors:** Y. Song, Z. Wang, Q. Tang, H. Xu, C. Xing, Z. Miao, C. Yang

**Affiliations:** * Department of Surgical Oncology and General Surgery, The First Hospital of China Medical University, Shenyang, China

**Keywords:** Superficial gastric cancer, cancerous ulcer, Borrmann type iv

## Abstract

In this report, we describe a patient presenting with the superficial spreading type of early gastric cancer (egc) accompanied by cancerous ulcers. Disease progression and treatment outcome are discussed. After symptoms persisted for more than 1 year, the patient underwent total gastrectomy with D2 lymph node dissection. The patient was diagnosed with superficial spreading cancer (ssc), accompanied by an extensive iic lesions. The progression of this patient suggests that the co-occurrence of cancerous ulcers may contribute to egc development to some extent. As is known, egc often develops into advanced gastric cancer with time. However, in our case, we observed a process during which partial cancerous changes developed into ssc over 18 months. Superficial spreading cancer should be considered an egc variant, which may have the ability to spread superficially along the stomach wall without invading the muscularis propria. But we speculate that, if gene expression changes for some reason, the malignant ssc cells may acquire the ability to grow deeply into the stomach wall. Eventually, Borrmann type iv gastric cancer may develop.

## 1. INTRODUCTION

Advances in diagnostic techniques since the late 1970s have led to an increased incidence of early gastric cancer (egc). A rare subtype of egc is superficial spreading cancer (ssc) of the stomach, which extends widely along the mucosa or submucosa. The present report describes a patient with ssc accompanied by cancerous ulcers; disease progression as observed by endoscopy and biopsy are discussed, and we propose a hypothesis for the evolution of this disease.

## 2. CASE REPORT

**FIGURE 1:**
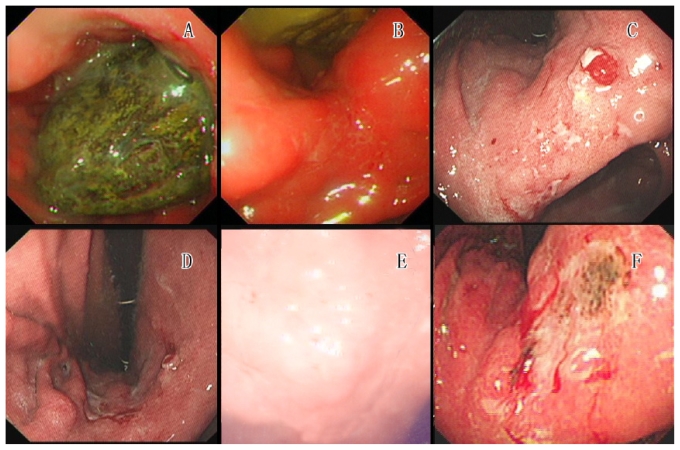
Upper gastrointestinal endoscopy results. (A) Solid, yellow-green bezoar, about 5×6 cm, located in the gastric fundus. (B) Superficial ulcerations measuring 10 mm in the greatest dimension; marked congestion and edema in the circumambient mucosa in the gastric angle. (C) Sporadic superficial ulcerations measuring 5 mm in the greatest dimension, flat, with white pustules. The mucosa showed marked congestion and edema in the gastric angle. (D) Sporadic superficial ulcerations measuring 3 mm in the greatest dimension. Rough mucosa is present in the stomach angle. (E) Reddish ulceration measuring 0.8×0.6 cm, the bottom covered with blood crust, boundaries obscure. Marked congestion and edema were present in the circumambient rough mucosa in the gastric angle. (F) A large area of mucosa (measuring about 3.5×4.0 cm) in the lesser curvature of the gastric body and gastric angle, showing congestion and edema with areas of erosion. We also found sporadic superficial ulcerations, with some white pustules and blood crust, brittle.

A 56-year-old man was admitted to hospital because of progressively severe upper abdominal pain. At 18 months before admission, this patient felt sudden upper abdominal pain after excessive ingestion of haws (a medicinal fruit of the *Crataegus* species). The patient tested positive for a *Helicobacter pylori* infection by ^13^C urea breath test, and a subsequent upper gastrointestinal endoscopy revealed a solid yellow-green bezoar [Figure 1(A)], with superficial ulceration in the circumambient mucosa of the gastric angle [Figure 1(B)].

**FIGURE 2:**
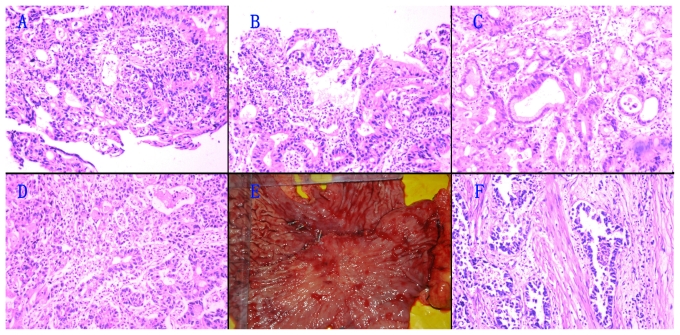
Pathology findings. Hematoxylin and eosin staining, 100× magnification. (A,B) Chronic severe gastritis, showing surface erosion, moderate-to-severe epithelial dysplasia, and partial cancerous changes. (C) Moderate-to-severe epithelial dysplasia and partial cancerous changes. (D) Moderately differentiated tubular adenocarcinoma. (E) Resected specimen showing early gastric cancer of the superficial spreading type (extensive iic). (F) Moderately differentiated tubular adenocarcinoma.

Two days later, an endoscopic lithotripsy was performed. During that procedure, sporadic flat superficial ulcerations in the gastric angle [Figure 1(C)] were noted. To evaluate the therapeutic efficacy of the endoscopic lithotripsy, endoscopy was performed again 2 days later. That endoscopy revealed sporadic superficial ulcerations in the gastric angle [Figure 1(D)]. Photomicrographs of the endoscopic biopsy specimen revealed chronic severe gastritis, surface erosion, and moderate-to-severe epithelial dysplasia, indicating cancerous changes [Figure 2(A,B)]. Surgery was therefore advised, but the patient refused for personal reasons and was discharged.

Abdominal pain recurred intermittently in the following months, and 1 year before readmission, an upper gastrointestinal endoscopy revealed a reddish ulceration—0.8×0.6 cm, the bottom covered with blood crust—with obscure boundaries, marked congestion, and edema in the circumambient rough mucosa in the gastric angle [Figure 1(E)]. Photomicrographs of the endoscopic biopsy specimen from the ulceration revealed moderate-to-severe epithelia dysplasia suggesting cancerous changes [Figure 2(C)]. The patient still refused therapeutic surgery.

One month before admission, the patient experienced progressively severe abdominal pain. He was referred to us for further therapy. Upper gastrointestinal endoscopy revealed a large area (3.5×4.0 cm) of congested mucosa, erosion, and edema in the lesser curvature of the gastric body and gastric angle. We also found sporadic superficial ulcerations, with some white pustules and blood crust [Figure 1(F)]. A photomicrograph of the endoscopic biopsy specimen from the ulceration revealed moderately differentiated tubular adenocarcinoma [Figure 2(D)]. The patient underwent total gastrectomy with D2 lymph node dissection. The abdominal pain completely subsided, and the patient was discharged.

As shown in Figure 2(E), the patient was diagnosed with superficial spreading egc accompanied by an extensive iic macroscopic lesions. The pathology examination revealed a moderately differentiated tubular adenocarcinoma, with 0 of 13 lymph nodes positive, and no vascular or lymphatic invasion [Figure 2(F)]. The tumour had invaded the submucosa; the proximal, distal, radial margins were free of tumour. Immunohistochemical analyses for p53 and Bcl2 protein on paraffin-embedded tissue were both positive.

## 3. DISCUSSION

### 3.1 Is There a Relationship Between the Cancer and the Ulcers?

From the photomicrographs in Figure 1(B–D), we observed that the area of gastric ulceration gradually decreased. However, 6 months later, the area of severe ulceration had increased. The last endoscopic evaluation revealed severe ulceration. The pathology examinations revealed progression from partial cancerous changes to superficial spreading egc. Throughout the process of gastric cancer progression, we observed cancerous ulcers. Is there a relationship between the cancer and the ulcers?

Several growth factors, including hepatocyte growth factor (hgf) and fibroblast growth factor β (fgfb) are involved in ulcer healing processes 1. Stromal cells and fibroblasts secrete hgf, which can accelerate gastric ulcer healing by the induction of gastric cell migration. The c*-met* proto-oncogene encodes the hgf receptor and is amplified in gastric cancer cells. Overexpression of hgf during the cancerous ulcer healing process could also stimulate the spread of gastric cancer cells. Fibroblast growth factor β is released at sites of tissue damage and tissue remodelling by several proteases. It has a broad spectrum of activity, including stimulation of protease production and cell migration. The stimulatory signals induced by the growth factors involved in the ulcer healing process may also result in migration and extracellular matrix degradation around gastric cancer cells, meeting the prerequisite for cancer invasion and metastasis. Thus, the presence of ulcers may contribute to egc development to some extent. However, it is not clear why the superficial spreading lesion does not invade the muscularis propria during progression.

### 3.2 Why Does the Lesion Fail to Invade the Muscularis Propria During Progression?

The five pathology examinations [Figure 2(A–D,F)] reveal progression from partial cancerous changes to ssc over 18 months. As is known, egc often develops into advanced gastric cancer with time. However, in the present case, we observed partial cancerous changes developing into ssc. Why does the lesion fail to invade the muscularis propria during progression?

Most gastric cancers—and other solid gastrointestinal cancers—usually grow deeply into the stomach wall in a three-dimensional manner. It is very interesting to observe that ssc spreads superficially along the stomach wall, but does not penetrate into the muscularis propria. Although the incidence of ssc is relatively low in China, we have investigated 79 cases of ssc (pers. obs.). Among those cases, the largest lesions were between 3×7.5 cm and 12×11.5 cm. Although the lesions are diffuse and lack a distinct margin, all have been confined to the mucosa or submucosa.

Several studies have described low expression of epidermal growth factor, transforming growth factor β, and fgfb in the malignant ssc cells 2,3, which may inhibit the penetrating growth of the malignant cells. Tomoda *et al.* 4 detected lower microvessel density and less vascular endothelial growth factor in ssc than in the penetrating type of gastric cancer. Haraguchi *et al.* 5 studied the relationship between growth patterns and dna ploidy and found that the malignant cells of ssc showed lower dna ploidy more often than did the penetrating type. Some gastric carcinomas with low dna ploidy and weak secretion of vascular endothelial growth factor may spread widely in the mucosa without vertical invasion through the gastric wall 6. These findings imply a biologically mild nature and may explain the excellent prognosis associated with surgery. There is a possibility that even large superficial spreading lesions are not able to invade into the muscularis propria.

We therefore propose the hypothesis that ssc should be considered an egc variant that possesses unique features different from those of other types of egc. The malignant ssc cells may have the ability to spread superficially along the stomach wall and not to invade into the muscularis propria. But if gene expression changes for some reason, will ssc develop?

In our investigations, we have found that most ssc lesions invade the submucosa (56 of 79). Borrmann type iv is known to be a diffusely infiltrating cancer with lack of a distinct margin 7. It often invades deeply into the entire gastric wall along the submucosa. Is there a relationship between ssc and Borrmann type iv?

We speculate that the malignant ssc cells have the ability to spread superficially along the stomach wall but not to invade the muscularis propria. However, if gene expression changes, they may acquire the ability to grow deeply into the stomach wall. Eventually, the scc may develop into Borrmann type iv. Though no literature about this hypothesis was available, we may be able to compare the protein expression profile for the two diseases by two-dimensional gel electrophoresis. Then, we may be able to identify the target genes by plasmid vector construction and gene transfection.

In the current case, immunohistochemical analyses were positive for p53 and Bcl2 protein, which is consistent with other results in the literature 8,9. Early gastric cancers with low levels of apoptosis, increased Bcl2, and high levels of p53 expression are more likely to invade and metastasize 8. Maybe p53 and Bcl2 could be considered candidate genes. Furthermore, if the molecular mechanism of this process can be identified—specifically, if the major site that effects the change of gene expression can be found, it could be considered a molecular marker that would help to make clinical predictions or early clinical diagnoses of Borrmann type iv gastric cancer. Moreover, our theory may suggest a promising future therapeutic strategy based on targeted therapy against Borrmann type iv gastric cancer.

## 4. CONCLUSIONS

This report describes the presence of cancerous ulcers during progression of egc in a single patient. Further molecular and biologic studies are required to clarify the role of the cancerous ulcers in the morphogenesis and growth mechanisms of ssc.

The growth mechanisms of ssc are still not clear. Can ssc develop into Borrmann type iv gastric cancer? Future studies are required to more completely outline a mechanism.
